# Physical Activity Types and Programs Recommended by Primary Care Providers Treating Adults With Arthritis, DocStyles 2018

**DOI:** 10.5888/pcd18.210194

**Published:** 2021-10-14

**Authors:** Dana Guglielmo, Kristina A. Theis, Louise B. Murphy, Michael A. Boring, Charles G. Helmick, John D. Omura, Erica L. Odom, Lindsey M. Duca, Janet B. Croft

**Affiliations:** 1 Division of Population Health, National Center for Chronic Disease Prevention and Health Promotion, Centers for Disease Control and Prevention, Atlanta, Georgia; 2 Oak Ridge Institute for Science and Education, Oak Ridge, Tennessee; 3 Optum Life Sciences, Inc, Eden Prairie, Minnesota; 4 ASRT Inc, Smyrna, Georgia; 5 Division of Nutrition, Physical Activity, and Obesity, National Center for Chronic Disease Prevention and Health Promotion, Centers for Disease Control and Prevention, Atlanta, Georgia; 6 Epidemic Intelligence Service, National Center for Chronic Disease Prevention and Health Promotion, Centers for Disease Control and Prevention, Atlanta, Georgia

## Abstract

Primary care providers (PCPs) can offer counseling to adults with arthritis on physical activity, which can reduce pain and improve physical function, mental health, and numerous other health outcomes. We analyzed cross-sectional 2018 DocStyles data for 1,366 PCPs who reported they always or sometimes recommend physical activity to adults with arthritis. Most PCPs sampled (88.2%) recommended walking, swimming, or cycling; 65.5% did not recommend any evidence-based, arthritis-appropriate physical activity programs recognized by the Centers for Disease Control and Prevention. Opportunities exist for public health awareness campaigns to educate PCPs about evidence-based physical activity programs proven to optimize health for adults with arthritis when more than counseling is needed.

**Summary**

**What is already known on this topic?**

Primary care providers (PCPs) are instrumental in promoting physical activity among adults with arthritis. Although specific guidance can maximize counseling effectiveness, little is known about what PCPs recommend to patients.

**What is added by this report?**

Among PCPs recommending physical activity to adults with arthritis, 88.2% recommended low-impact activities (walking, swimming, or cycling); 65.5% did not recommend any arthritis-appropriate physical activity programs recognized by the Centers for Disease Control and Prevention. Nearly 80% of PCPs not recommending these programs attributed it to being unaware of them.

**What are the implications for public health practice?**

Public health awareness campaigns could strategically focus on promoting arthritis-appropriate physical activity programs to PCPs, which might ultimately increase their reach to adults with arthritis.

## Objective

Physical activity is recommended for adults with all types of arthritis because it can reduce pain and improve physical functioning, mood, and quality of life (1,2). Professional organizations encourage health care professionals to counsel adults with arthritis on physical activity and recommend supervised programs when needed (3,4). Primary care providers (PCPs) frequently treat arthritis (5) and are instrumental in promoting physical activity. Although we previously found that 98.4% of PCPs always or sometimes recommend physical activity to adults with arthritis (6), the content of physical activity counseling may affect its effectiveness (3). Addressing patient concerns (eg, arthritis-specific physical activity barriers such as pain) warrants specific guidance and referrals to safe, supervised programs (3). To build on a previous study, we examined physical activity types and programs recommended among PCPs recommending physical activity to adults with any type of arthritis and compared distributions of characteristics of PCPs recommending programs versus PCPs unaware of them.

## Methods

We analyzed cross-sectional data from 2018 Porter Novelli DocStyles (https://styles.porternovelli.com/docstyles), an online national market research survey assessing PCP attitudes, patient encounters, and use of medical information resources. Eligible DocStyles participants were family practitioners, internists, obstetrician/gynecologists, and nurse practitioners aged 21 or older, living and practicing in the US, practicing for at least 3 years, treating at least 10 patients weekly, and working at an individual, group, or inpatient practice. From June through August 2018, Porter Novelli invited participants by email to complete the survey from the Sermo Global Medical Panel (www.sermo.com), SurveyHealthcareGlobus (www.surveyhealthcareglobus.com), and WebMD (www.webmd.com). Target quotas (1,000 PCPs, 250 obstetricians/gynecologists, and 250 nurse practitioners) were met by inviting highly responsive participants (defined as completing >75% of any kind of survey [not only DocStyles] in which they had been invited to participate) first from among those not participating in DocStyles 2017. Of 2,582 invited persons, 1,505 completed the survey (response rate, 58.3%) and were compensated $55 to $77 based on number of questions asked. We excluded 116 PCPs not treating adults with arthritis and 23 never recommending physical activity, which resulted in an analytic sample of 1,366. Additional survey details are available elsewhere (6). Although analyses were not subject to Centers for Disease Control and Prevention’s (CDC’s) institutional review board, we followed all Council of American Survey Research Organizations guidelines, and the data set was deidentified.

The 2018 DocStyles Survey included a module with questions about recommendations for CDC-recognized arthritis-appropriate physical activity programs (hereafter “programs”) (7), which have an evidence base for addressing physical activity barriers (8). PCPs treating an average of at least 1 adult with arthritis weekly completed multiple choice questions about physical activity counseling for adults with arthritis, including physical activity types, programs recommended, and reasons for not recommending programs.

We calculated percentages for physical activity type and program variables overall (N = 1,366) and reasons for not recommending programs among PCPs not recommending programs (n = 895). To identify opportunities for promoting program awareness, we generated distributions of PCP characteristics overall (N = 1,366) and for those recommending programs (n = 471) and unaware of programs (n = 710). We generated percentages using SAS version 9.4 (SAS Institute Inc); we performed χ^2^ tests in Excel version 2008 (Microsoft Corp) to assess differences (significant at ɑ = .05) between PCP groups.

## Results

PCPs were commonly aged 50 or older (46.2%; 95% CI, 43.5%−48.8%), men (57.5%; 95% CI, 54.8%−60.1%), non-Hispanic White (67.1%; 95% CI, 64.6%−69.6%), and working in a group outpatient practice (67.5%; 95% CI, 65.0%−70.0%) (Table). Most PCPs recommended walking, swimming, or cycling (88.2%; 95% CI, 86.5%−89.9%), stretching (63.8%; 95% CI, 61.3%−66.4%), and physical therapy (60.8%; 95% CI, 58.2%−63.4%) (Figure). Programs were recommended less frequently than physical activity: 34.5% (n = 471) of PCPs recommended 1 or more programs. The most commonly recommended programs were the Arthritis Foundation’s Aquatic Program (18.0%; 95% CI, 16.0%−20.0%), the Arthritis Foundation’s Exercise Program (14.4%; 95% CI, 12.6%−16.3%), and Walk With Ease (13.8%; 95% CI, 12.0%−15.7%) (Figure). Most PCPs did not recommend any programs (65.5%; 95% CI, 63.0%−68.0%); among this group (n = 895), the most commonly reported reasons were being unaware of them (n = 710; 79.3%; 95% CI, 76.7%−82.0%); programs were unavailable in their area (22.5%; 95% CI, 19.7%−25.2%), unaffordable for patients (12.5%; 95% CI, 10.3%−14.7%), or inaccessible to patients (12.2%; 95% CI, 10.0%−14.3%); and believing patients would not attend (10.5%; 95% CI, 8.5%−12.5%).

**Table:** Distribution of Characteristics of Primary Care Providers Who Recommend Physical Activity to Adults With Arthritis,^a^ Overall and by Counseling Subgroups, DocStyles 2018

Characteristic	Overall Sample (N = 1,366)		PCPs Recommending Physical Activity Programs^b^ (n = 471)		PCPs Unaware of Physical Activity Programs^c^ (n = 710)		PCPs Recommending vs Unaware χ^2 ^ *P* Value^d^
	n	%^e^ (95% CI)	n	%^e^ (95% CI)	n	%^e^ (95% CI)	
**Sociodemographic**							
**Age group, y**							
21−39	285	20.9 (18.7−23.0)	105	22.3 (18.5−26.1)	158	22.3 (19.2−25.3)	.96
40−49	450	32.9 (30.4−35.4)	157	33.3 (29.1−37.6)	228	32.1 (28.7−35.6)	
≥50	631	46.2 (43.5−48.8)	209	44.4 (39.9−48.9)	324	45.6 (42.0−49.3)	
**Sex**							
Male	785	57.5 (54.8−60.1)	268	56.9 (52.4−61.4)	407	57.3 (53.7−61.0)	.94
Female	581	42.5 (39.9−45.2)	203	43.1 (38.6−47.6)	303	42.7 (39.0−46.3)	
**Race or ethnicity**							
Non-Hispanic White	917	67.1 (64.6−69.6)	260	55.2 (50.7−59.7)	532	74.9 (71.7−78.1)	<.001
Non-Hispanic Asian	260	19.0 (16.9−21.1)	123	26.1 (22.1−30.1)	105	14.8 (12.2−17.4)	
Other race or ethnicity	189	13.8 (12.0−15.7)	88	18.7 (15.2−22.2)	73	10.3 (8.0−12.5)	
**Region**							
Northeast	321	23.5 (21.2−25.8)	102	21.7 (17.9−25.4)	180	25.4 (22.1−28.6)	.71
Midwest	317	23.2 (21.0−25.4)	103	21.9 (18.1−25.6)	170	23.9 (20.8−27.1)	
South	471	34.5 (32.0−37.0)	170	36.1 (31.8−40.4)	233	32.8 (29.4−36.3)	
West	257	18.8 (16.7−20.9)	96	20.4 (16.7−24.0)	127	17.9 (15.1−20.7)	

**Medical Practice**							
**Provider type**							
Family practitioner	477	34.9 (32.4−37.5)	160	34.0 (29.7−38.3)	243	34.2 (30.7−37.7)	.16
Internist	503	36.8 (34.3−39.4)	200	42.5 (38.0−46.9)	235	33.1 (29.6−36.6)	
Obstetrician/gynecologist	173	12.7 (10.9−14.4)	50	10.6 (7.8−13.4)	103	14.5 (11.9−17.1)	
Nurse practitioner	213	15.6 (13.7−17.5)	61	13.0 (9.9−16.0)	129	18.2 (15.3−21.0)	
**Years practicing medicine**							
<10	287	21.0 (18.8−23.2)	99	21.0 (17.3−24.7)	164	23.1 (20.0−26.2)	.84
10−19	497	36.4 (33.8−38.9)	176	37.4 (33.0−41.7)	252	35.5 (32.0−39.0)	
20−29	389	28.5 (26.1−30.9)	140	29.7 (25.6−33.9)	194	27.3 (24.0−30.6)	
≥30	193	14.1 (12.3−16.0)	56	11.9 (9.0−14.8)	100	14.1 (11.5−16.6)	
**Privileges at a teaching hospital**							
Yes	623	45.6 (43.0−48.3)	253	53.7 (49.2−58.2)	302	42.5 (38.9−46.2)	.02
No	743	54.4 (51.7−57.0)	218	46.3 (41.8−50.8)	408	57.5 (53.8−61.1)	
**Main work setting**							
Individual outpatient practice	298	21.8 (19.6−24.0)	102	21.7 (17.9−25.4)	145	20.4 (17.5−23.4)	.92
Group outpatient practice	922	67.5 (65.0−70.0)	317	67.3 (63.1−71.5)	491	69.2 (65.8−72.6)	
Inpatient practice	146	10.7 (9.0−12.3)	52	11.0 (8.2−13.9)	74	10.4 (8.2−12.7)	
**Average number of patients treated per week**							
<75	279	20.4 (18.3−22.6)	61	13.0 (9.9−16.0)	184	25.9 (22.7−29.1)	<.001
75−99	281	20.6 (18.4−22.7)	90	19.1 (15.6−22.7)	158	22.3 (19.2−25.3)	
100−124	431	31.6 (29.1−34.0)	140	29.7 (25.6−33.9)	224	31.5 (28.1−35.0)	
≥125	375	27.5 (25.1−29.8)	180	38.2 (33.8−42.6)	144	20.3 (17.3−23.2)	
**Average number of adults with arthritis treated per week**							
1−9	589	43.1 (40.5−45.7)	161	34.2 (29.9−38.5)	346	48.7 (45.0−52.4)	.01
10−19	456	33.4 (30.9−35.9)	178	37.8 (33.4−42.2)	223	31.4 (28.0−34.8)	
≥20	321	23.5 (21.2−25.8)	132	28.0 (24.0−32.1)	141	19.9 (16.9−22.8)	
**Number of practitioners in practice^f^**							
1 or 2	304	22.3 (20.0−24.5)	94	20.0 (16.3−23.6)	151	21.3 (18.3−24.3)	.60
3−5	383	28.0 (25.7−30.4)	146	31.0 (26.8−35.2)	182	25.6 (22.4−28.9)	
6−11	303	22.2 (20.0−24.4)	109	23.1 (19.3−27.0)	161	22.7 (19.6−25.8)	
≥12	376	27.5 (25.2−29.9)	122	25.9 (21.9−29.9)	216	30.4 (27.0−33.8)	
**Patient portal available**							
Yes	986	72.2 (69.8−74.6)	349	74.1 (70.1−78.1)	520	73.2 (70.0−76.5)	.84
No or not sure	380	27.8 (25.4−30.2)	122	25.9 (21.9−29.9)	190	26.8 (23.5−30.0)	

Abbreviation: PCP, primary care provider.

a Main analytic sample (N = 1,366) were primary care providers who responded “always” or “sometimes” to “When you see patients with arthritis/rheumatic conditions how often do you recommend physical activity/exercise for management of their condition?”

b Defined using the question, “Have you ever recommended one or more of the following exercise programs to your patients? Select all that apply.” Answer options included 6 arthritis-appropriate physical activity programs recognized by the Centers for Disease Control and Prevention (Arthritis Foundation Exercise Program, Walk With Ease, Active Living Every Day, Fit & Strong!, EnhanceFitness, and Arthritis Foundation Aquatic Program) and none of these.

c Defined as responding “I do not know about these programs.” to “Why don’t you recommend the listed exercise programs to your patients with arthritis/rheumatic conditions? Select all that apply.”

d χ^2^ tests compared the percentage distributions of PCP characteristics for PCPs recommending physical activity programs and PCPs unaware of physical activity programs; significant if *P* < .05.

e Some columns do not sum to 100% because of rounding.

f Number of practitioners in the practice includes the respondent.

**Figure:**
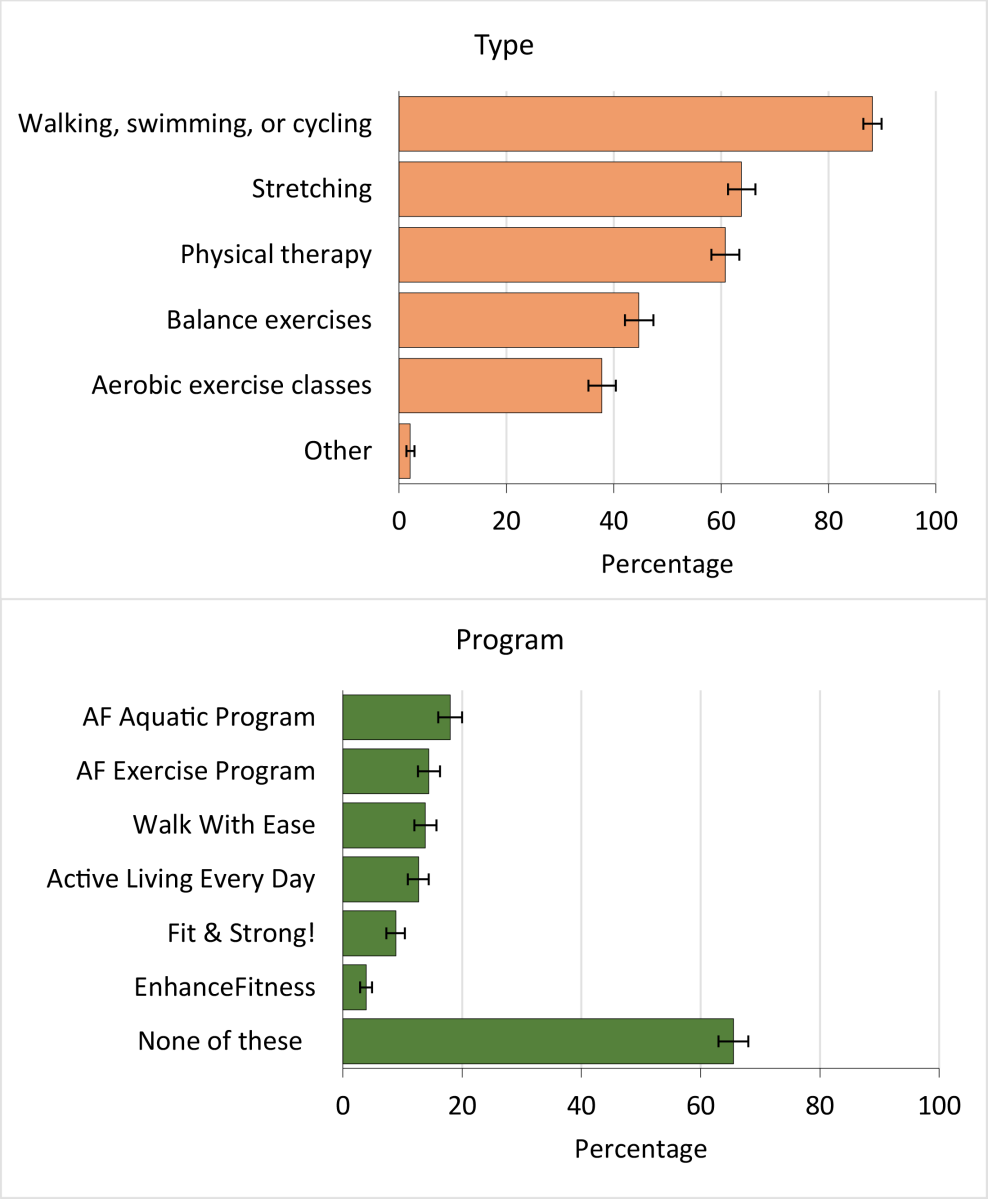
Physical activity types and programs recommended by primary care providers (N = 1,366) who recommended physical activity to adults with arthritis, DocStyles 2018. For physical activity types, survey participants were asked, “When you talk to your patients with arthritis/rheumatic conditions about physical activity/exercise what type of activity do you recommend? Select all that apply.” For physical activity programs, survey participants were asked, “Have you ever recommended one or more of the following exercise programs to your patients? Select all that apply.” Survey participants were primary care providers who responded “always” or “sometimes” to “When you see patients with arthritis/rheumatic conditions how often do you recommend physical activity/exercise for management of their condition?” Error bars indicate 95% CIs. Abbreviation: AF, Arthritis Foundation.

**Table:** 

Physical Activity	n	% (95% CI)
**Types recommended**		
Walking, swimming, or cycling	1,205	88.2 (86.5−89.9)
Stretching	872	63.8 (61.3−66.4)
Physical therapy	831	60.8 (58.2−63.4)
Balance exercises	611	44.7 (42.1−47.4)
Aerobic exercise classes	517	37.8 (35.3−40.4)
Other	29	2.1 (1.4−2.9)
**Arthritis-appropriate programs recommended**		
None of these	895	65.5 (63.0−68.0)
Arthritis Foundation Aquatic Program	246	18.0 (16.0−20.0)
Arthritis Foundation Exercise Program	197	14.4 (12.6−16.3)
Walk With Ease	189	13.8 (12.0−15.7)
Active Living Every Day	173	12.7 (10.9−14.4)
Fit & Strong!	121	8.9 (7.3−10.4)
EnhanceFitness	53	3.9 (2.9−4.9)

Overall, 34.5% (95% CI, 32.0%−37.0%) of PCPs reported recommending 1 or more arthritis-appropriate programs (Figure). The distribution of most characteristics did not differ significantly between PCPs recommending physical activity programs and those unaware of physical activity programs, including by age, sex, region, provider type, years practicing, main work setting, number of practitioners in practice, and patient portal availability (Table). Exceptions were race or ethnicity (*P* < .001), privileges at a teaching hospital (*P* = .02), average number of patients treated per week (*P* < .001), and average number of patients with arthritis treated per week (*P* = .01). Distributions for PCPs recommending versus not recommending programs were significantly different for these same 4 variables.

## Discussion

At least 3 in 5 PCPs recommending physical activity to adults with arthritis recommended low-impact aerobic activities (walking, swimming, or cycling), stretching, or physical therapy. These activities align with professional guidance on optimal activities for most adults with arthritis (2,3), although appropriate activities differ by individual. Still, most PCPs sampled (65.5%) did not recommend programs, with 79.3% of these PCPs unaware of them. Our study demonstrates that the guidance PCPs already consistently offer to patients can be strengthened by recommending programs when needed.

PCPs are important promoters of physical activity (4). Creating a safe, specific, and tailored exercise plan is important for adults with arthritis (2); many are hesitant about physical activity because of misplaced fears about joint damage (9). Additionally, adults with arthritis report the absence of referrals to programs from health care providers as a barrier to exercise; therefore, they are likely to be receptive to program referrals (10).

Adults with arthritis may benefit from greater awareness of safe, arthritis-appropriate, evidence-based physical activity programs. Proven program outcomes include improved physical activity levels, strength, and balance, and reduced pain, fatigue, and stiffness (11). PCPs aware of local resources could be more likely to provide referrals (12). Strategies to promote PCP awareness of physical activity programs include distributing information about program benefits and availability through clinical practice sites, health departments, public health partnerships, continuing medical education, clinical–community linkages, and electronic medical record prompts.

Study strengths include the large sample size and ability to assess counseling for arthritis management. Limitations include using an opportunity sample that was not nationally representative and survey questions that featured a limited list of physical activity types and programs. Future studies might consider examining additional activity and program recommendations.

Strategic focus of public health awareness campaigns promoting arthritis-appropriate physical activity programs to PCPs could increase their reach to adults with arthritis.
